# Neuroblastoma of the elderly, an oncologist’s nightmare: case presentation, literature review and SEER database analysis

**DOI:** 10.1186/2162-3619-3-20

**Published:** 2014-07-17

**Authors:** Elisa Rogowitz, Hani M Babiker, Mohammed Kanaan, Rebecca A Millius, Q Scott Ringenberg, Maria Bishop

**Affiliations:** 1 University of Arizona College of Medicine, 1501 N. Campbell Ave, Tucson, AZ 85724, USA; 2 Division of Hematology-Oncology, Department of Medicine, Southern Arizona VA Health Care System, 3601 S 6Th Ave, Tucson, AZ 85723, USA; 3 Division of Hematology-Oncology, Department of Medicine, University of Arizona College of Medicine, 1501 North Campbell Avenue, Tucson, AZ 85724, USA; 4 Division of Hematology-Oncology, Department of Medicine, University of Arizona Cancer Center, 1515 N Campbell Ave, Tucson, AZ 85724, USA; 5 Department of Pathology, University of Arizona College of Medicine, 1501 North Campbell Avenue, Tucson, AZ 85724, USA

**Keywords:** Thymus, Neuroblastoma, Adult, SIADH

## Abstract

Neuroblastoma is considered a pediatric malignancy as over 95% of cases are diagnosed in patients ≤10 years old. This cancer is extremely rare in elderly patients. We conducted a Surveillance, Epidemiology, and End Results (SEER) database analysis in the USA between 1973–2007 that revealed only 35 elderly patients (>60 years of age) with neuroblastoma of whom only 2 patients had primary mediastinal neuroblastoma. There is a paucity of treatment and survival outcomes data for the elderly owing to the rarity of neuroblastoma in this population. Currently there are no standard guidelines or protocols for treatment of adult neuroblastoma. We report a rare and challenging case of an 86-year old patient presenting with mediastinal neuroblastoma and syndrome of inappropriate antidiuretic hormone secretion (SIADH) successfully treated with resection. Herein, we also provide a review of the literature and updated survival data on neuroblastoma based on results of our SEER database review.

## Background

Neuroblastoma is the most common extracranial solid tumor among infants and children [1]. It is almost exclusively a pediatric neoplasm [2,3]: the median age at diagnosis is 2 years, and more than 90% of patients are diagnosed under ten years of age [1,3]. In patients over 30 years, it is rare (0.2 cases per million inhabitants per year) and its incidence becomes increasingly scarce in the elderly population [4]. Our review of literature revealed 35 elderly patients with neuroblastoma with only two prior cases of mediastinal neuroblastoma. Our 86-year-old patient is the oldest reported patient with mediastinal neuroblastoma.

Neuroblastoma is a type of neuroblastic carcinoma that can arise anywhere along the peripheral sympathetic nervous system. It was first described by Dr. Rudolf Virchow as a “glioma” in the abdominal cavity in 1864 [5]. By 1910, Dr. Homer-Wright determined that this tumor originates from primitive neural cells based on the histological rosettes it forms within the bone marrow [6].

Histologically, neuroblastomas display a diverse spectrum of differentiation. In well-differentiated tumors, cells are approximately 7 to 10 microns in diameter, have hyperchromatic nuclei and scant cytoplasm, and may form Homer-Wright rosettes. These rosettes are characterized by a central lumen filled with neuropil, a delicate fibrillary material consisting of neuronal processes, which are surrounded by a halo of tumor cells [7]. Given that Homer-Wright rosettes are also found in medulloblastomas and peripheral primitive neuroectodermal tumors (PNET), it is believed that their presence indicates neuronal differentiation [7]. Yet, the cellular mechanisms responsible for the formation of rosettes are not fully understood. Undifferentiated neuroblastoma tumors are composed almost entirely of neuroblasts with very few Schwannian (or stromal) cells and are difficult to distinguish from other small round blue cell tumors on light microscopy. Neuroblastomas typically react with antibodies that distinguish neural tissue (e.g., neuron-specific enolase, neuropil, synaptophysin, chromogranin, and S100).

Neuroblastomas have a very broad spectrum of clinical behavior, which can range from spontaneous regression, to benign ganglioneuroma, or to aggressive disease with metastatic dissemination leading to death. This diversity correlates closely with numerous clinical and biological factors (including patient age, tumor stage and histology, and genetic abnormalities). One of the most important prognostic factors is age at diagnosis. In the pediatric population, the younger the child, the better is the prognosis. Neuroblastomas in newborns often remit spontaneously. Children over 5 years old have an unfavorable prognosis [8]. The natural history in adult and elderly populations is unknown, given the rare incidence in these groups. Polychemotherapy is the standard treatment in children; however, there are no standard chemotherapy protocols in elderly patients. In addition, review of case reports and case series of neuroblastoma in elderly patients indicate a different biology with a more indolent behavior in comparison to children and adolescent patients [1-3]. Herein, we present a very rare case of neuroblastoma in an elderly patient treated with surgical resection alone.

### Presentation

An 86-year-old Hispanic male veteran presented to the emergency room with fatigue, feeling “shaky” for five days, and shortness of breath. His only medication was amlodipine for hypertension, and his family history was significant for a mother who died of lung cancer.

**Figure 1:**
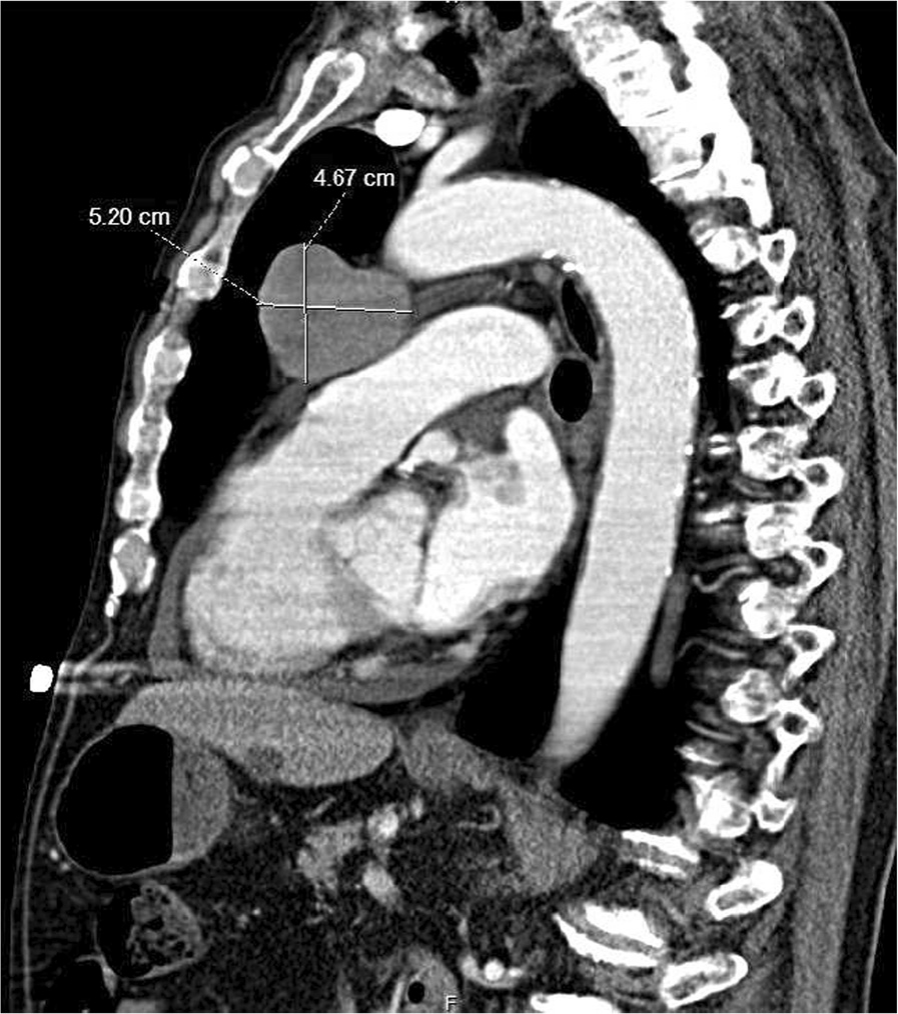
**CT scan of the chest.** Sagittal CT scan image of the chest reveals a large mediastinal mass measuring 5.2 × 4.7 cm. There is no hilar or mediastinal lymphadenopathy.

**Figure 2:**
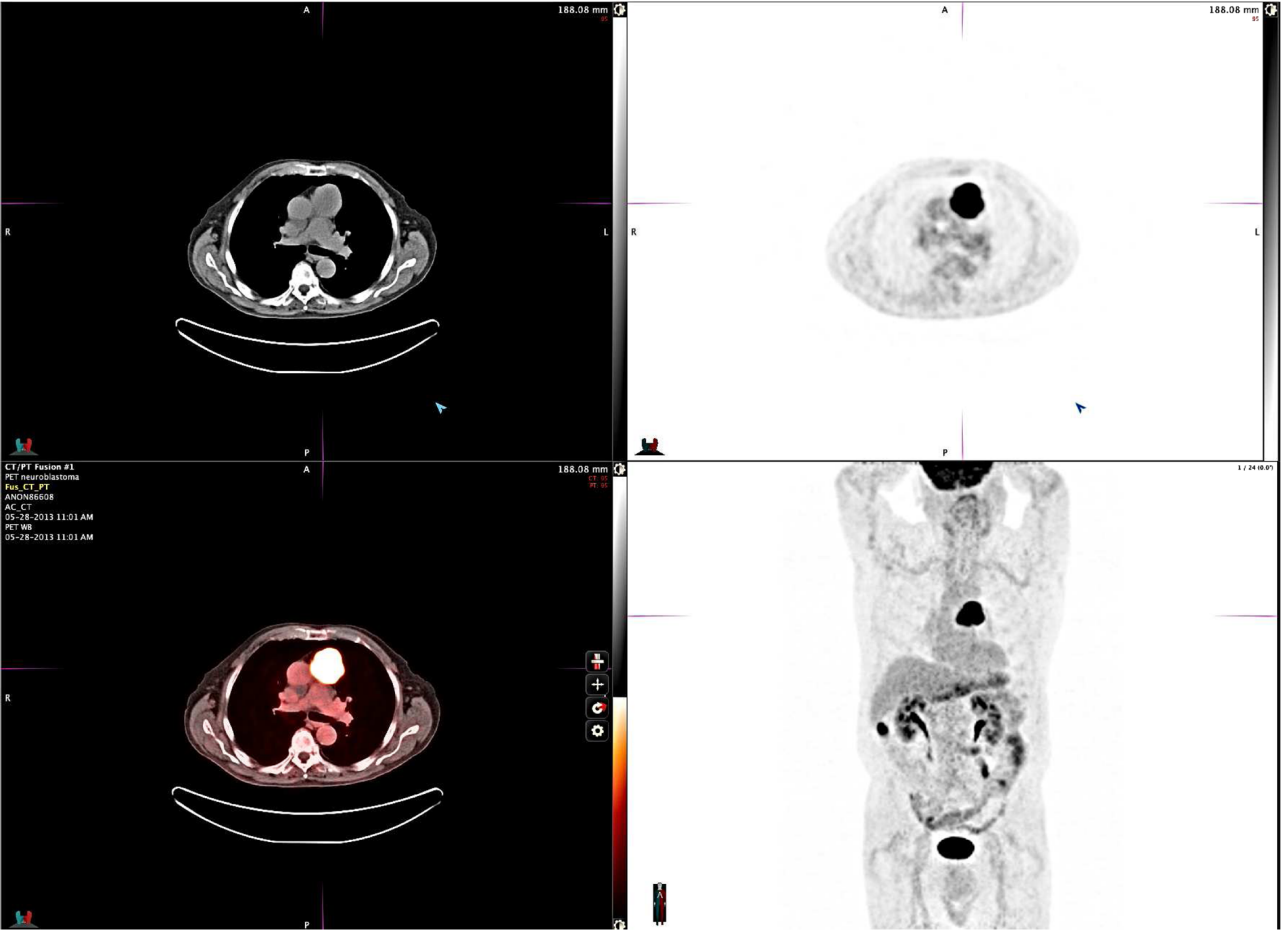
**Body PET scan pre-surgery.** PET scan of the chest and body reveals an anterior mediastinal mass measuring 5.0 × 5.1 cm with an SUV of 15. No additional suspicious masses or adenopathy are appreciated. There is an area of increased metabolic activity in the inferior aspect of the right hepatic lobe that is related to bowel activity.

**Figure 3:**
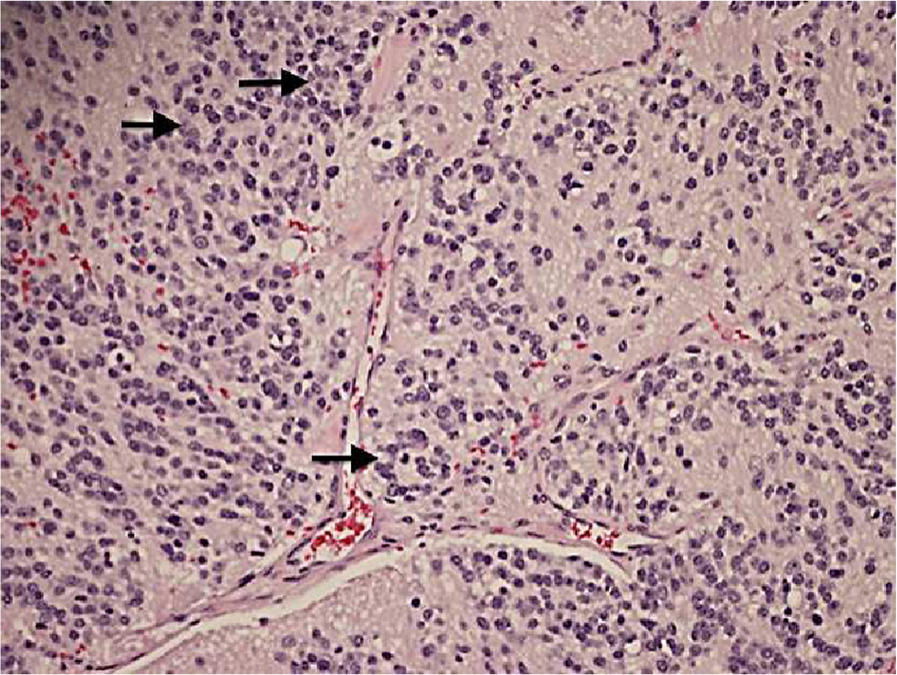
**Resected mediastinal mass surgical pathology.** Hemotoxylin & Eosin stained section of resected mediastinal mass reveals scattered small Homer-Wright rosettes (arrows): small dark neuroblastoma cells in circular groups around pale fibrillary neuropil (200× magnification).

**Figure 4:**
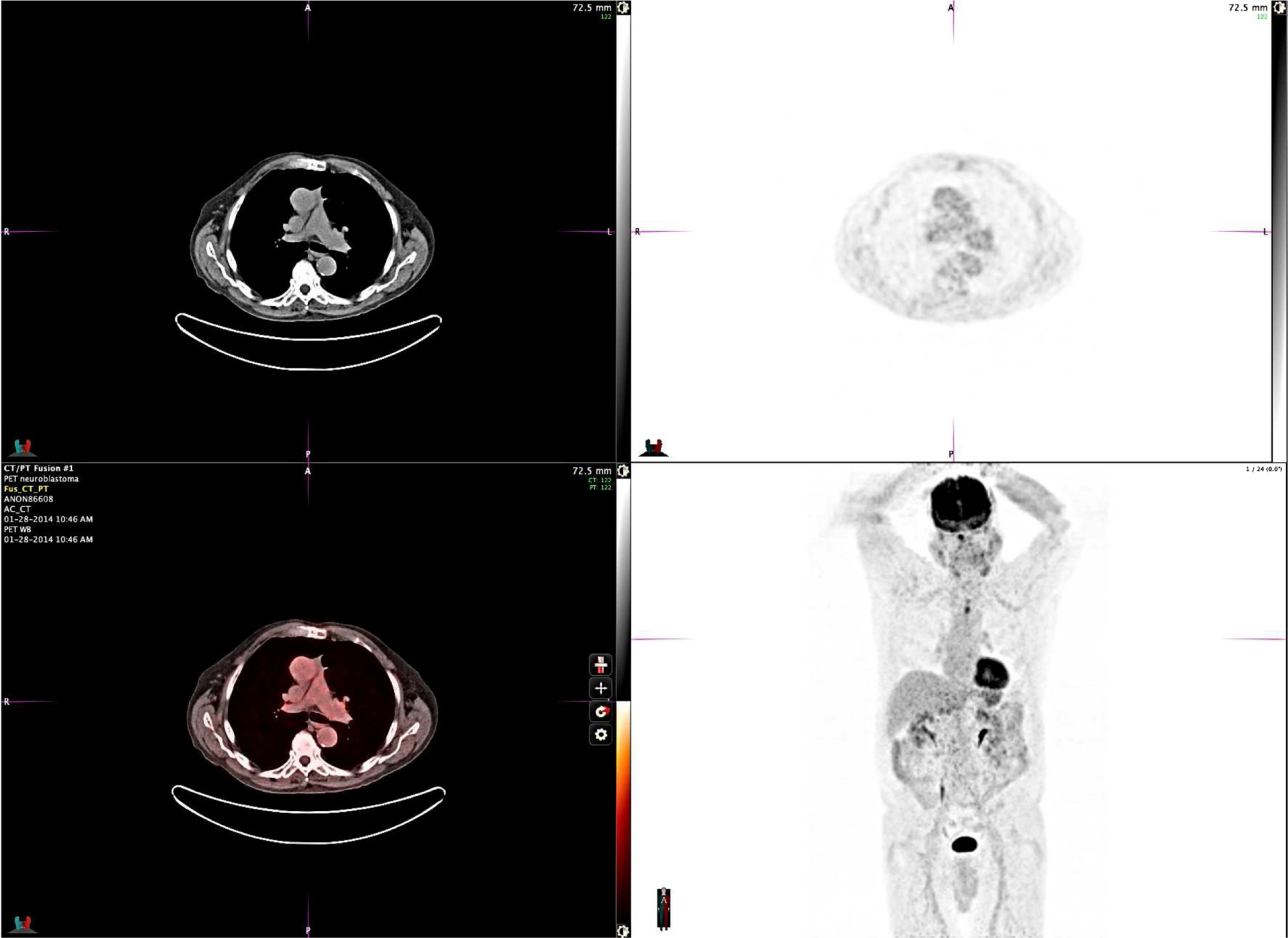
**Body PET scan post-surgery.** PET scan of the chest and body performed 7 months after surgical resection reveals no foci of abnormal FDG avid activity within the chest, no pulmonary nodules, and normal cardiomediastinal silhouette appearance.

In the emergency room, his vital signs were stable and his physical exam was only remarkable for minimally reactive, asymmetric pupils. Lab studies disclosed sodium of 128 mEq/L (reference range 135-145 mEq/L), plasma osmolality of 266 mOsm/kg (284-306 mOsm/kg), urine osmolality of 533 mOsm/kg (250-900 mOsm/kg), a urinary sodium concentration of 103 mEq/L (40–220 mEq/l/24 hours), and an alkaline phosphatase of 151 U/L (40–125 U/L). Results of all other laboratory studies were within normal ranges. A chest X-ray demonstrated an enlarged mediastinum. Follow-up contrast-enhanced chest computed tomography (CT) revealed an anterior mediastinal mass measuring 5.2 × 4.7 cm in the sagittal section (Figure 1). Staging contrast CT scan of his head and abdomen/pelvis were negative. The patient was diagnosed with SIADH and was placed on fluid restriction, which led to improvement of his sodium levels and abatement of his symptoms. CT-guided fine needle aspiration biopsy was performed and pathology revealed a poorly differentiated neuroendocrine carcinoma. Immunohistochemistry showed positive staining for synaptophysin and chromogranin, focal positivity for MAK6 (pankeratin), and absence of S-100. Based on the morphology and immunoprofile, this tumor could either represent a primary thymic neuroendocrine carcinoma or metastatic large cell neuroendocrine carcinoma of pulmonary origin. A whole-body ^18^F-FDG-PET/CT scan demonstrated the anterior mediastinal mass, measuring 5.0 × 5.1 cm with an SUVmax of 15 (Figure 2). No additional pulmonary masses, suspicious osseous lesions, or adenopathy were appreciated.He was referred to cardiothoracic surgery and underwent a sternotomy, “en bloc” thymectomy, and wedge left pulmonary resection, all with negative margins. Surgical pathology revealed a tan-red thymus with a firm encapsulated nodule measuring 6.5 × 5.5 cm and areas of hemorrhage. Microscopic extracapsular extension was present with perithymic adipose infiltration and capsular adherence to the pleura and pericardium, but lung tissue and lymph nodes were not involved. The tumor cells had a neuroendocrine appearance with abundant neutropil, formed rosettes, and a low mitotic index (<2% of cells) (Figure 3). Immunohistochemical staining revealed positive neuroendocrine markers: CD56, synaptophysin and chromogranin in tumor cells, neurofilament protein in neuropil, S-100 (focally), and negative cytokeratin AE1/AE3 and GFAP. This pattern is typical for neuroblastomas. The absence of cytokeratins excluded a diagnosis of thymic carcinoma. The abundant presence of neuropil and the absence of clear-cut membrane staining with CD99 were sufficient to exclude PNET. Furthermore, the interphase fluorescence in situ hybridization (FISH) was negative for EWSR1 t(11;22)(q24;q12) translocation, excluding Ewing’s sarcoma and supporting the diagnosis of neuroblastoma. According to the revised International Neuroblastoma Staging System (INSS), our patient has a stage 1 tumor.The patient tolerated the surgical procedure without complications and his sodium levels remained normal without fluid restriction following surgery. Repeat PET scan 7 months after treatment indicated no mediastinal mass or residual disease (Figure 4). The patient remains free of disease and asymptomatic 11 months after resection.

## Discussion

### Epidemiology

We obtained and analyzed neuroblastoma incidence from the U.S. National Cancer Institute’s Surveillance, Epidemiology, and End-Results registry (SEER). All neuroblastoma cases diagnosed between 1973 and 2010 were selected, yielding 3818 patients between the ages of 0 and 103 years. The data was categorized according to demographic information, anatomic site, and interval disease-specific survival (DSS) collected for up to 60 months. Patient age at diagnosis was classified into three groups: <18 years, 18–60 years, and >60 years. Within the oldest group, data were analyzed by gender, race, anatomic location, and 5-year DSS.

The SEER database included 3602 pediatric, 181 adult, and 35 elderly patients (>60 years of age), representing 94.3%, 4.7%, and 0.9% of neuroblastoma cases, respectively (Table 1). In elderly patients, 15 patients were male (43%) and 20 were female (57%). Thirty patients were identified as White (86%), 3 patients as Native American, Asian, or Pacific Islander (8.6%), and 2 patients as Black (5.7%). The most common primary sites in patients > 60 years of age were soft tissue including the heart (60%), trachea and respiratory organs including the mediastinum (31.4%), and the testis (5.7%). Only 2 of the 3818 patients (0.05%) were reported to be over 60 years old with a mediastinal tumor site. Thus, our patient is extremely unique. He is the third patient with these characteristics and is the eldest of them.

**Table 1:** SEER database analysis of neuroblastoma patient characteristics between 1973–2010 according to age distribution

		**<18 years**	**18-60 years**	**>60 years**
**Number of patients**		3602	181	**35**
**Gender (%)**	Male	53%	51%	**43%**
	Female	47%	49%	**57%**
**Race (%)**	White	81%	77%	**86%**
	Black	12%	15%	**5.7%**
	Other	7%	8%	**8.6%**
**MCPS (%)**	1^ST^ MCPS	Endocrine including Thymus (42.9%)	Central Nervous System (39%)	**Soft tissue including heart (60%)**
	2^nd^ MCPS	Soft Tissue including heart (23.6%)	Retroperitonium (17%)	**Trachea, mediastinum and respiratory organs (31.4%)**
	3^rd^ MCPS	Retroperitonium (12.4%)	Endocrine including Thymus (14%)	**Testis (5.7%)**
	4^TH^ MCPS	Trachea, Mediastinum and Respiratory Organs (9.5%)	Soft Tissue including heart (7.7%)	**Stomach (2.9%)**
**5 year DSS**		46.9%	32.6%	**40%**

MCPS: Most Common Primary Site; DSS: Disease specific survival.

Bold data highlights SEER data base analysis results in patients older than 60 years of age.

### Clinical presentation

The clinical presentation of neuroblastoma reflects the tumor’s primary location and extent of metastatic disease, if present. Patients with localized disease can be asymptomatic whereas patients with advanced disease can appear systemically ill. Based on our SEER data, neuroblastomas most frequently originate in endocrine tissues including the adrenal glands, soft tissues, and the retroperitoneum. These tumors metastasize to bone marrow (70.5%), bone (55.7%), lymph nodes (30.9%), liver (29.6%), intracranial and orbital sites (18.2), lung (3.3%), and the central nervous system (0.6%) [9]. The sites of the primary tumor and metastases correlate with the most common signs and symptoms, which include weight loss, abdominal pain and distention, back pain, weakness, spinal compression, anemia, and hypertension [9]. Horner’s syndrome is described with cervical sympathetic ganglia involvement. Children are more likely than the elderly to present with widespread and severe symptoms, as pediatric neuroblastomas metastasize frequently and rapidly. Additionally, 40% of childhood neuroblastomas secrete catecholamines (homovanillic acid, vanillylmandelic acid, or dopamine), which can cause hypertension [2]. Multiple studies suggest that adult patients rarely have positive urine catecholamine tests [2,10].

Our 86-year old patient with Stage 1 mediastinal neuroblastoma presented with vague complaints of weakness, shakiness, and weight loss with moderate hyponatremia. His SIADH resolved after the tumor’s excision, suggesting a paraneoplastic etiology. Interestingly, a paraneoplastic SIADH syndrome has been described in 83% of mediastinal and thymic neuroblastomas in elderly patients whereas few of the other cancers occurring in the mediastinum secrete ectopic ADH [10-13]. A review of the literature revealed five elderly thymic neuroblastoma patients, ages 60–80, with proven paraneoplastic SIADH [10,11,13]. Of these, one patient presented similarly to our patient with weakness, memory loss, and severe hyponatremia [10]. Another had symptoms of SIADH and coronary artery disease [13]. Both had resolution of SIADH following resection. The other cases of neuroblastoma were otherwise asymptomatic. Although neuroblastomas in elderly patients are exceedingly rare, they should be considered as a diagnostic possibility. This is especially true for patients with SIADH and a mediastinal mass.

### Prognosis

Neuroblastomas have a very broad spectrum of clinical behavior, which can include spontaneous regression, maturation to a benign ganglioneuroma, or aggressive disease with metastatic dissemination leading to death. The clinical array correlates closely with numerous clinical and biological factors. The International Neuroblastoma Pathology Classification system, developed in 1999, evaluates prognosis of childhood neuroendocrine tumors based on the patient’s age at diagnosis and morphology (characterization of stroma, grade of differentiation, and mitosis-karyorrhexis index [MKI]) [8]. The most important prognostic factor for this system is age at diagnosis. It awards the best prognosis to newborns, followed by infants, then toddlers. Children over age 5 are given an unfavorable prognosis regardless of histology. If this tool is applied to our 86-year old patient, it yields contrasting results. Our patient’s overall five-year survival would be estimated at 0% even though his pathology (schwannian stroma-poor morphology, cellular differentiation falling in the continuum between poorly differentiated and differentiated subtypes, and low MKI of <2%) would confer a favorable prognosis [8]. Based on this, we conclude that this classification system cannot be used reliably in older patients.

Limited prognostic data exists for adolescents, adults, and the elderly. However, it is clear that the natural history of neuroblastoma of the elderly differs from that of children. In these populations, the cancer has more indolent behavior, but carries a worse prognosis [2]. This is most likely attributable to the multiple comorbidities of this population and the concomitant inability to tolerate aggressive systemic therapy. Although mediastinal neuroblastoma appears to have a better prognosis in the elderly in comparison to other groups according to our review and published case reports, the rarity of this disease precludes a survival analysis in a clinical trial. Cytologic atypia and high mitotic activity were correlated with a more aggressive tumor behavior by a retrospective case series of 22 adult and elderly patients with thymic neuroblastoma [1].

Our SEER database analysis also demonstrates that elderly patients have a different disease course than children. We observed that all ages of neuroblastoma patients with any tumor location have a 5-year DSS of 40%. Pediatric patients have a 5-year DSS of 46.9%. In the elderly, the 5-year DSS was lower at 40%. However, patients with mediastinal, tracheal, and other respiratory neuroblastomas had improved outcomes and the elderly fared better. Of 353 patients with neuroblastoma at these sites between the ages of 0–73, the 5-year DSS was 66% for all ages, 67% for the 342 pediatric patients, 44% for the 9 adult patients, and 100% in the 2 elderly patients. Despite the extremely limited data of the elderly with mediastinal neuroblastomas, the survival difference between the pediatric and elderly groups concurs with the proposal of a more prolonged tumor progression in the elderly [2]. Another study reported that adult and elderly patients had a longer interval from symptom onset to diagnoses and from recurrence/progression to death [3]. This effect may be attributed to the rarity of metastasis or infrequency of MYCN gene amplification in older patients [12,14].

### Treatment strategies and survival data

Neuroblastoma treatment in pediatric populations is well-studied but remains complex and difficult. Surgical resection is the mainstay of treatment in low-risk patients, with optional combination chemotherapy including cyclophosphamide, carboplatin, cisplatin, etoposide, teniposide, and doxorubicin. Infants can be observed for spontaneous remission or progression. Radiotherapy is recommended only if the disease progresses despite surgery and chemotherapy. For high-risk pediatric patients, an aggressive combined modality approach, including surgical resection, high-dose chemotherapy with stem cell rescue, radiation therapy and biologic/immunologic therapy has shown improved long-term outcomes [15]. Nevertheless, most high-risk patients eventually relapse and die of their disease.

In a Memorial-Sloan Kettering retrospective review of 30 patients with advanced neuroblastoma, aged 12–41 at diagnosis, high-dose induction chemotherapy followed by surgery was shown to achieve a minimal disease state in over 50% of patients [1]. Additionally they found that: 1) 3F8 immunotherapy is helpful in chemo-resistant neuroblastoma; 2) moderate doses of local radiation (21 Gy) aid in achieving local control; 3) standard-dose chemotherapy only has a palliative role; and 4) anti-GD2 antibodies, cis-retinoic acid, oral etoposide, and topotecan provide the basis of a multi-modality approach and improve the poor prognosis.

Another group performed a retrospective review of 27 adolescent and adult neuroblastoma patients who did not have MYCN oncogene amplification. This population ranged from 12–69 years of age at diagnosis, although the distribution was skewed towards the younger end of this range, with a median age of 17 years. They used surgery alone for stage I tumors and surgery followed by radiotherapy for stage II disease. Stage III patients were given induction therapy with 6 cycles of neoadjuvant cisplatin plus etoposide alternated with adriamycin, cyclophosphamide and vincristine, followed by either surgery or 35–45 Gy radiotherapy; they then underwent consolidation with hemi-body irradiation and four cycles of the alternating chemotherapy regimen [3]. For stage IV, the protocol for stage III was used, with the subsequent addition of intensified chemotherapy and radiotherapy protocols. They found 5-year OS rates of 83% and 28% for patients staged I-II and III-IV, respectively. Additionally, elderly patients had a longer interval between recurrence or progression and death, which they speculate is due to the lack of MYCN amplification. This mutation occurs in 25-40% of high-risk pediatric tumors and correlates with rapidly progressive tumor behavior [14,16]. Interestingly, the vast majority of adult neuroblastomas lack the MYCN amplification [1-3,12,14].

Currently, there is a paucity of treatment and survival outcome data for the elderly owing to the rarity of neuroblastoma in this population. There are no standard treatment guidelines or chemotherapy protocols. Expert opinion suggests surgical tumor resection. When surgical margins and lymph nodes are negative, as in our patient, this approach can result in long-term relapse-free survival. Several case reports and case series reported patients as disease-free up to 108 months after surgical tumor excision [2,3,10-12]. The intense pediatric protocols are poorly tolerated by elderly patients. Discontinuing those protocols most likely contributed to the improved survival trend of elderly neuroblastoma patients over the past decade as observed by a SEER database review of patients diagnosed between 1973–2010 [4]. Hence, surgical resection is the main treatment and adjuvant chemotherapy or radiotherapy is reserved for advanced stage disease.

## Conclusion

Mediastinal neuroblastoma in elderly patients is a very rare disease with sparse data available in the literature pertaining to the epidemiology, treatment, and outcomes. Early stage mediastinal neuroblastoma in elderly patients can be managed with surgical resection alone, due to the fact that this disease has a different biology and more indolent behavior than is observed in pediatric patients. In addition, elderly patients cannot tolerate aggressive chemotherapy regimens offered to pediatric patients. Several case reports indicated a disease-specific survival ranging from 12 to 108 months in this population after surgical resection. Herein we report a case of the oldest patient with early stage mediastinal neuroblastoma successfully treated with surgical resection alone.

## Consent

Written informed consent was obtained from the patient for publication of this case report and the accompanying images. A copy of the written consent is available for review by the Editor-in-Chief of this journal.

## Abbreviations

SEER: Surveillance, Epidemiology, and End Results; SIADH: Syndrome of inappropriate antidiuretic hormone secretion; PNET: Peripheral primitive neuroectodermal tumors; FISH: Fluorescence in situ hybridization; DSS: Disease-specific survival; INSS: International neuroblastoma staging system.

## Competing interests

The authors declare that they have no competing interests.

## Authors’ contributions

All authors have contributed significantly to the study and preparation of the manuscript and have approved the final version. EAR and HMB provided equal contribution towards publication of the manuscript.
